# Viscoelastic Properties of Epoxidized Natural Rubber/Poly(lactic acid) PLA/ENR Blends Containing Glycidyl-POSS and Trisilanolisooctyl-POSS as Functional Additives

**DOI:** 10.3390/ma14102686

**Published:** 2021-05-20

**Authors:** Magdalena Lipińska, Klaudia Toczek, Magdalena Stefaniak

**Affiliations:** Institute of Polymer and Dye Technology, Lodz University of Technology, 90-924 Lodz, Poland; klaudia.toczek@dokt.p.lodz.pl (K.T.); magdalenastefaniak95@gmail.com (M.S.)

**Keywords:** thermoplastic vulcanizates, poly(lactic acid)/epoxidized natural rubber blends, polyhedral oligomeric silsesquioxanes POSS, viscoelastic properties

## Abstract

The glycidyl-POSS (Polyhedral Oligomeric Silsesquioxanes, Polysilsesquioxane, POSS) (Gly-POSS) and trisilanolisooctyl-POSS (HO-POSS) were applied as functional additives influencing on the viscoelastic properties of the dynamic vulcanized PLA/ENR (poly(lactic acid)/epoxidized natural rubber) blends. The plasticizing effect of HO-POSS on PLA/ENR melt, leading to the decrease of complex viscosity at 160 °C, was observed. After the incorporation of Gly-POSS into PLA/ENR blends the complex viscosity increased confirming that the epoxy groups of Gly-POSS were able to react with the functional groups of ENR and the groups present at the end of PLA chains. The incorporation of Gly-POSS into 40:60 PLA/ENR blend provided significant enhancement of the storage shear modulus G’ at 30 °C. Furthermore, the glass transition temperatures T_g_ of ENR phase for PLA/ENR/Gly-POSS blends were shifted to higher values of temperature as compared with blends modified by HO-POSS. Strong reduction of the elongation at break E_b_ for 40:60 PLA/ENR/Gly-POSS blend indicated that Gly-POSS particles acted as multifunctional cross-links reducing elasticity of the material. The modification of 40:60 PLA/ENR blend by HO-POSS molecules led to lower values of composting coefficient K_C_ indicating stronger deterioration of the mechanical properties that resulted from more intense degradation processes occurring during disposal in soil.

## 1. Introduction

Thermoplastic elastomers (TPEs) belong to a class of polymeric materials that can be processed in a molten state like thermoplastic polymers and possess elastic and mechanical properties similar to crosslinked rubber. Dynamic vulcanization (DV) is a process that allows to produce thermoplastic vulcanizates (TPVs) [1] a special type of TPEs. The most commonly used thermoplastic vulcanizates TPVs are based on thermoplastic elastomer blends such as: polystyrene (PS) and styrene-butadiene rubber (SBR) [2], polypropylene (PP), and ethylene-propylene-diene rubber (EPDM) [3] blends. The vulcanization of rubber and the formation of crosslinks takes place during reactive melt mixing (DV) of PP/EPDM leading to a two-phase morphology in which elastomer phase is dispersed in a melt-processable thermoplastics matrix [3]. The partial curing is a factor leading to the stabilization of the blend morphology. Typically, traditional industrial TPVs are obtained using petroleum-based thermoplastic additives. Environmental concerns are forcing scientific research into finding other types, more ecological thermoplastic vulcanizates. TPVs based on polyethylene and reclaimed rubber were reported due to their waste management’s benefits [1,4]. The efforts of scientists are currently focused on creating a thermoplastic vulcanizate obtained by dynamic vulcanization that can be classified as a more environmentally friendly material containing a lower content of petroleum-based components and higher content of components obtained from renewable resources. In our work, we prepared by physical blending elastic material based on two components poly(lactic acid) (PLA) and the epoxidized natural rubber (ENR). Both components are classified as renewable resources materials. Hence, the environmental impact of the final product is decreased in comparison to traditionally used PP/EPDM thermoplastic vulcanizates (TPVs) in industry [3].

The incorporation into rubber as an additive the biodegradable polyesters offers promising environmentally friendly opportunity to create rubber blends.

Poly(Lactic acid) (PLA) is a thermoplastic material having biodegradability and compostability as compared with non-degradable fossil-based polymers [5,6]. It is classified as a bio-based material that is obtained from renewable resources such as sugarcane or corn starch [7]. This strategy, the use of PLA derived from biomass, removes the petrochemical component from the TPV product’s life cycle. However, PLA based TPV materials still offer only limited recyclability connected with hydrolytic degradation of PLA phase. Especially if the bonds formed in second rubber phase are covalent.

Poly(lactic acid) is one of the most readily degradable thermoplastic materials. The factors influencing on the mechanism and speed of PLA degradation under various environmental conditions were analyzed by many authors [8,9,10,11,12,13,14,15]. The main mechanism of PLA degradation is hydrolytic degradation or enzymatic degradation. The hydrolysis occurs via autocatalytic random chain scission reactions in which the ester bonds are broken down [12]. The formed carboxyl end groups were found to self-catalyze the hydrolysis reaction [12]. The molecular and supramolecular structure of PLA affects the degree and rate of degradation [8,10]. The most important factor influencing on PLA degradation carried out under natural and laboratory conditions are: the amount of D-lactide isomer in chain structure [13] and the degree of PLA crystallinity [14]. The rate of PLA hydrolysis is also strongly affected by the degradation conditions such as: temperature, humidity, and pH [9,11,15]. Moliner et al. [11] reported for PLA samples after immersion during 30 days at room temperature in various aqueous solutions at different pH the limited hydrolysis of the ester bonds in PLA and the formation of few free alcoholic and carboxylic groups. In real conditions and in natural environment or in the landfills the hydrolytic degradation of PLA structure can last up to many weeks. Among other factors, the weathering conditions, soil composition, and the presence of microorganisms influence the PLA degradation time. Hence, the biodegradation of PLA in a compost environment was studied [16,17]. Abiotic hydrolysis was suggested as a main depolymerization mechanism in which the first step is the reduction of the polymer molecular weight. Furthermore, the low molecular weight oligomers and monomers are assimilated by microorganisms [16]. Pantani et al. [16] reported that initial morphology and crystallinity degree achieved after processing strongly affected the further degradation steps during composting. The crystallinity reduced the swelling of the samples preventing the enzymatic attach and oligomer diffusion [16]. In another [17] study, four composting commercial practices (turned windrow, anaerobic digestion followed by static pile, static pile, in-vessel followed by roofed windrow) were used to estimate the disintegration of commercial PLA products. The disintegration rates of foodware and packaging based on PLA were affected by the material structure as well as by the composting method.

The elevated temperature, oxygen, and mechanical stress during PLA processing also cause the thermo-oxidative and thermomechanical degradation of PLA leading to random chain scission and the formation of reactive terminal carbonyl and hydroxyl groups [18]. Speranza et al. [19] investigated the degradation of commercial grade PLA in the molten state by analyzing the viscosity during the dynamic rheological tests. The significant reduction of complex viscosity η* was observed confirming the decrease of polymer molecular weight due to chain scissions [19]. Cuadri et al. [18] studied the effect of thermomechanical degradation in presence of oxygen using dynamic oscillatory rheometer. The reduction in viscoelastic properties and the decrease of storage G’ modulus and loss G’’ modulus were observed due to chain scission phenomena as the applied degradation time was longer [18]. Moreover, the thermo-oxidative and thermomechanical degradation of PLA led to the loss of thermomechanical resistance of the material [18].

In addition to the degradability, PLA shows comparable mechanical properties, stiffness and tensile strength to polystyrene (PS), polyethylene (PE), and polypropylene (PP) [20], that makes it useful substitute of above mentioned polymers in TPVs. The PLA/natural rubber (NR) [21,22,23], PLA/acrylate rubber [24], PLA/nitrile rubber [25], PLA/elastomeric ethylene-butyl acrylate-glycidyl methacrylate terpolymer (EBA-GMA) [26] blends were obtained.

From an environmental point of view, the use of epoxidized modification of natural rubber as the rubber phase in TPVs seems promising. Natural rubber (NR) belongs to biobased materials extracted from natural resource, rubber trees (*Hevea brasiliensis*), in the latex form [21]. The thermal degradation of ENR starts at about 300 °C, and the degradation leads to the transformation of the epoxy group and the formation of aldehydic compound with the chain breakdown [27]. The formation of carbonyl in the epoxy backbone as a result of thermo-oxidation was also reported for amine epoxy [28]. The presence of an oxirane ring in the natural rubber creates the possibility of ENR curing by compounds such as amino acids derivatives or flavonoids containing hydroxyl groups [29]. The chemical interactions between epoxy functional groups and the reactive terminal PLA groups are also possible. Some authors [30] reported that mechanical properties of PLA/ENR blends were enhanced as compared with PLA/NR blends. The PLA/NR blends showed phase separated morphology, the better blend compatibility was observed for PLA/ENR. It was attributed to chemical bonds formed between oxirane ring of ENR and hydroxyl groups of PLA [31]. Zheng et al. [32] also confirmed grafting of the PLA chains on ENR network during dynamic vulcanization of PLA/ENR blend. The additives like cellulose nanocrystals were additionally applied to enhance mechanical properties of PLA/ENR blends [33].

We propose to apply the polyhedral oligomeric silsesquioxanes POSS as an additive into PLA/ENR blends. The POSS particles are the smallest organosilica molecules in which the cubic inorganic cage (consisting of Si and O backbone) is linked with various functional side groups [34]. POSS nanoparticles are added to various polymers to enhance mechanical properties, thermal stability, and oxidation resistance [34,35]. Polyhedral oligomeric silsesquioxanes such as octavinyl-POSS [36,37], octaisobutyl-POSS [38], octa (3-chloropropyl)-POSS [39], aminopropylisobutyl-POSS [40] were used as a functional additive to PLA that influenced on its mechanical, thermal, and morphological properties. Silsesquioxanes with reactive functional groups are able to undergo surface bonding, grafting, or polymerization. POSS particles with hydroxyl functional groups were adopted as initiators of lactide ring opening polymerization (ROP), hybrid structures containing the poly(lactic acid) chains tethered with POSS core were synthetized [41,42]. POSS with glycidyl functional groups was applied as a curing additive for the carboxyl-terminated poly(acrylonitrile-co-butadiene) [43]. The compatibilizing effect of epoxycyclohexyl-POSS on the poly (lactic acid)/poly(butylene succinate-co-adipate) PLA/PBSA blend was reported due to chemical reaction between epoxy group of POSS and the groups present at the end of PLA/PBSA chains [44].

The aim of our work was to create a flexible material, dynamically vulcanized, based on components from renewable sources. Using the melt-compounding method, poly(lactic acid)/epoxidize natural rubber PLA/ENR blends differing in the PLA content were obtained. The rheological characteristics of the PLA/ENR blends have been deeply investigated with an oscillating rheometer, both in the molten state and at room temperature. In particular, the influence of the thermo-oxidative processes and self-curing at various times of compression molding on viscoelastic properties was analyzed. These experiments provide valuable knowledge regarding the development and application of dynamic vulcanized PLA/ENR blends in industry.

The glycidyl POSS and trisilanolisobutyl-POSS were incorporated as additives to PLA/ENR blends. The presence of oxirane rings gives the possibility of reaction with functional groups of both PLA and ENR phase enhancing curing of the material.

This way the glycidyl-POSS influences on the static and dynamic mechanical properties of PLA/ENR blend. Various POSS molecules can be additives that are able to accelerate or postpone the thermal degradation of PLA leading to the changes of viscoelastic properties. The complex viscosity, storage shear modulus G’, and loss shear modulus G’’ of POSS modified PLA/ENR blend were analyzed. The effect of POSS incorporation on the degradation of the material and its mechanical properties after composting was estimated.

## 2. Materials and Methods

### 2.1. Materials and Preparation of the Blends

To prepare PLA/ENR blends, the ENR50 (Epoxyprene 50, Muang Mai Guthrie Company) the epoxidized natural rubber together with L-polylactide PLA, (CAS number 331335-50-1 product of Simagchem Corporation, China) were applied. Before the preparation of the blends, PLA pellets were dried at 80 °C for 24 h. The formulations of the blends were as follows: 20 g of PLA–80 g of ENR, further denoted as 20:80 PLA/ENR blend; 30 g of PLA–70 g of ENR, further denoted as 30:70 PLA/ENR blend; 40 g of PLA–60 g of ENR, further denoted as 40:60 PLA/ENR blend.

**Scheme 1:**
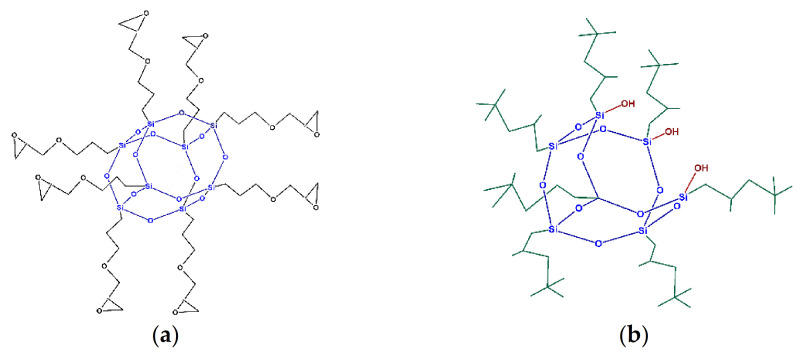
The structures of used polyhedral oligomeric silsesquioxanes POSS additives: (**a**)—glycidyl-POSS, (**b**)—trisilanolisooctyl-POSS.

A glycidyl-POSS cage mixture (EP0409 Hybrid Plastics, Hattiesburg, MS, USA, with eight glycidyl functional groups and epoxy equivalent weight 167), further denoted as Gly-POSS and trisilanolisooctyl-POSS (SO1455 Hybrid Plastics, Hattiesburg, MS, USA, an open cage POSS with seven isooctyl groups and three hydroxyl groups), further denoted as HO-POSS were added at 3 phr (parts per 100 g of the blend) to the 20:80 PLA/ENR and 40:60 PLA/ENR blend. The chemical structures of used POSS additives are showed at Scheme 1.

Blends were prepared by melt-mixing method using laboratory mixer with counter-rotating rotors (Brabender Lab Station Plasti-Corder, Duisburg, Germany). For this purpose, the weighted amount of PLA pellets was placed in heated (160 °C) mixer chamber and melted. Then appropriate amount of cut ENR rubber was added to molten PLA and mixed during 5 min at 150 r/min of rotor speed. In case of POSS modified PLA/ENR blends the weighted amount of Gly-POSS or HO-POSS was added to PLA/ENR and mixed during additional 5 min. Then, the compositions were removed from mixer and cooled. Prepared blends were compression molded to form films using hydraulic press at 200 bar and 160 °C during 15 or 30 min.

### 2.2. Viscoelastic Properties at 160 °C and at 30 °C

The dynamic viscoelastic behavior of PLA/ENR blends in melt state and at ambient temperature were studied using oscillation rheometer Ares G2 (TA Instruments) equipped with parallel plate-plate geometry (diameter of 25 mm). The following measurements were done: (a) time sweep test at 160 °C and at constant values of angular frequency and oscillation strain, equivalent of 1 s^−1^ oscillation strain rate, the freshly prepared samples were used for these tests; (b) frequency sweep tests at 160 °C, at constant value 1% of oscillation strain (linear viscoelastic region) and angular frequency range 0.3–628 rad s^−1^, the freshly prepared samples and samples after 15 and 30 min of compression molding were used for these tests; (c) frequency sweep tests at ambient 30 °C temperature, at constant value of oscillation strain 0.02% (linear viscoelastic region) and angular frequency range 0.3–628 rad s^−1^, samples after 15 min of compression molding were used for these tests; (d) amplitude oscillation tests at ambient 30 °C temperature, at constant values 10 rad s^−1^ (1.59 Hz) and 125.6 rad s^−1^ (20 Hz) of angular frequency and oscillation strain in range of 0.01–100%. The parameters such as: complex viscosity η* (Pa⋅s), storage shear modulus G’ (kPa), loss shear modulus G’’ (kPa) were registered.

### 2.3. Glass Transition Temperature T_g_ Studies

Glass transition temperatures of ENR phase in PLA/ENR blends were calculated from the oscillation viscoelastic measurements (ISO 6721-11:2019) of loss tan δ as a function of temperature in range of −30–25 °C, at constant angular frequency of 5 rad s^−1^ and at constant oscillation strain of 0.02% using Ares G2. DSC analysis was used to identify the glass transition temperatures of PLA phases in PLA/ENR blends. DSC tests were performed using DSC1 equipment (Mettler Toledo, Columbus, OH, USA). Tests were done under nitrogen atmosphere, two heating steps from 20 to 210 °C (heating rate 10 °C⋅min^−1^) were applied, the first heating step was to eliminate the heat history of the sample. The DSC glass transition measurements were done according to ISO 22768:2020 standard.

### 2.4. Thermogravimetric Analysis TGA

The thermal decomposition of layered double hydroxides was performed using TGA/DSC1 (Mettler Toledo) analyzer. Samples were heating from 25 to 800 °C in an argon atmosphere with the heating rate of 10 °C min^−1^.

### 2.5. Tensile Mechanical Properties Studies

The static tensile mechanical properties at room temperature were measured using universal testing machine Zwick Roell 1435 according to ISO 37:2005. The moduli at 100% elongation, tensile strength, and elongation at break were registered. Crosshead speed of 500 mm min^−1^ was used. Tests were done for six different testing type 2 dumb-bell specimens and the average value for each formulation was reported.

### 2.6. Degradation of the Material in Soil

The mechanical properties after degradation in soil during one month under controlled composting conditions (temperature 50 °C, humidity 90%, pH = 5.5) were determined similar according to ISO 37:2005. The soil ageing coefficient was calculated according to the Equation (1)

(1) $$K_{c}=\frac{{\left(TS*\;E_{b}\;\right)}_{composted}}{{\left(TS*\;E_{b}\right)}_{before\;composting}}$$

where *TS* is measured tensile strength and *E_b_* is measured elongation at break, respectively before and after composting.

### 2.7. Morphology of the Blend

The blend morphology was analyzed using SEM microscope (ISO 22493:2014) (JEOL 5500, Sendai, Japan) for the fracture surface covered by gold.

## 3. Results and Discussion

### 3.1. Viscoelastic Behavior of PLA/ENR Blends in Melt State

**Figure 1:**
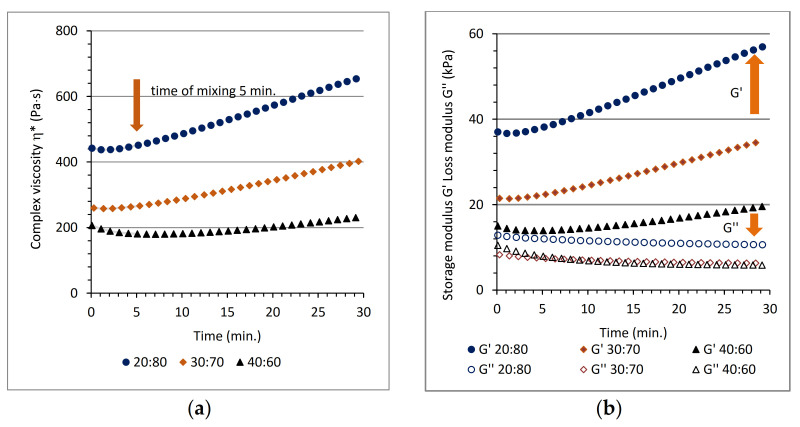
The complex viscosity η* as a function of time at processing temperature 160 °C at constant strain and angular frequency, equivalent of 1 s^−1^ oscillation strain rate (**a**). The storage modulus G’ and loss modulus G’’ as a function of time at processing temperature of 160 °C (**b**).

The important issue related to the processing of poly(lactic acid)/epoxidized natural rubber PLA/ENR blends is the phenomenon of thermo-oxidative degradation occurring together with interphase curing of both blend components. Melt properties of the mixed PLA/ENR material can be changed due to both: the chains scissions of PLA as well as the chemical reactions between two phases of the blend or in ENR phase. These interphase crosslinking reactions can occur between the functional end-chain groups of PLA and epoxy functionalities of ENR rubber. The viscoelastic behavior of PLA/ENR blends at processing temperature 160 °C was analyzed to estimate the curing and degradation effects in molten state. The values of complex viscosity η* and storage G’ and loss G’’ shear modulus as a function of time were analyzed (Figure 1).

The complex viscosity η* of the neat epoxidized natural rubber ENR at the beginning of the test was higher (6270 Pa s) and over 5 min decreased to the value of 3618 Pa s due to the effect of the temperature. No significant increase in the values of η*, G’ were observed after 10^th^ minute of measurement, and the values were adequately: the complex viscosity η* = 3423 ± 53 Pa.s; storage modulus G’ = 39.2 ± 0.4 kPa; loss modulus G’’ = 25.2 ± 0.3 kPa.

The higher content of PLA in PLA/ENR reduced the complex viscosity η* of the melt system. For all PLA/ENR blends up to 5 min of mixing the complex viscosity η* slightly decreased indicating melting of the system and probably no changes in the structure of blend components. At longer time, the end-chain groups of PLA generated after the chain scission were able to form covalent bonds with functional, epoxy groups of ENR leading to increase of the η*. After 15 and 30 min under conditions of measurements (1 s^−1^ oscillation strain rate, temperature 160 °C, presence of oxygen) the increase of complex viscosity due to the self-curing of the PLA/ENR material was adequately: 19% (15 min) and 50% (30 min) for 20:80 PLA/ENR. For 40:60 PLA/ENR blend less significant changes of complex viscosity occurred, and after 30 min of test the 6% increase of complex viscosity, due to the self-curing of the material, was observed. The partial curing of elastic phase influenced on the viscoelastic properties of blends in melt state. Storage modulus G’ increased and loss modulus G’’ decreased as the time of thermo-oxidative degradation was longer (Figure 1). The loss tan δ during 30 min of ageing decreased respectively: for 20:80 PLA/ENR blend from tan δ = 0.35 to 0.19; for 30:70 PLA/ENR blend from tan δ = 0.39 to 0.18; for 40:60 PLA/ENR blend from tan δ = 0.70 to 0.30. The changes of loss tan δ can be additional confirmation of the cross-linking reactions occurred between two phases of the blend during thermo-oxidative ageing.

**Figure 2:**
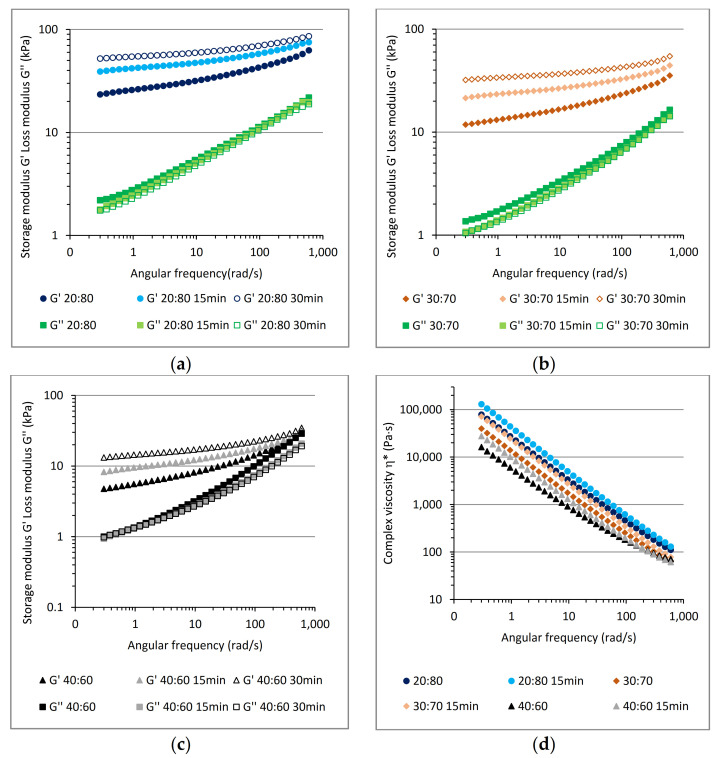
Influence of thermal treatment during 15 and 30 min of compression molding at 160 °C on the viscoelastic properties of molten PLA/ENR formulations, the storage modulus G’, loss modulus G’’ for: 20:80 PLA/ENR (**a**); 30:70 PLA/ENR (**b**); 40:60 PLA/ENR (**c**); and complex viscosity η* of PLA/ENR blends (**d**).

Due to the presence of thermoplastic PLA component the PLA/ENR blends can be repeatedly melted and processed at higher temperature by compression molding. To estimate the changes in material structure during compression molding at various time (15, 30 min) the viscoelastic properties as a function of angular frequency of the once again molten material were studied. The Figure 2 shows the values of storage shear modulus G’ and loss shear modulus G’’ measured at 160 °C for freshly mixed over 5 min. PLA/ENR blends and blends that were after mixing cooling and molded again in hydraulic press at 160 °C during 15 and 30 min. During compression molding the changes in the structure of the blend occurred that was confirmed by the viscoelastic properties of the molten material (Figure 2).

No significant effect of thermal treatment during 15 and 30 min on the virgin ENR was observed (Supporting Information Figures S1 and S2).

The higher amount of incorporated PLA reduced storage shear modulus G’ and complex viscosity η* of PLA/ENR blends. It should be marked that for all blends formulations the values of storage modulus G’ were higher than the values of loss modulus G’’ indicating the predominant elastic behavior of molten material in whole range of frequency studied (Figure 2). The storage modulus G’ for samples after thermal treatment during 15 and 30 min increased because of formed chemical bonds between ENR and PLA phase at interphase during compression molding. Effect was stronger after first 15 min of thermal treatment generating higher increase of G’. The increases of ∆G’ at 0.3 rad s^−1^ were calculated as: ∆G’ = G’_time = 15−_G’_time =_ 0, and were adequately: 20:80 PLA/ENR ∆G’= 15.6 kPa; 30:70 PLA/ENR ∆G’ = 9.5 kPa; 40:60 PLA/ENR ∆G’ = 3.5 kPa. During compression molding, stronger curing effect was generated for the blend of higher ratio of ENR to PLA. It could be explained by statistically higher amount of functional epoxy groups in similar volume in case of 20:80 PLA/ENR blend as compared with 40:60 PLA/ENR blend. It gave higher possibility of the reaction between epoxy groups of ENR chains and PLA end-chains. The values of loss modulus G’’ for molten samples decreased after thermal treatment during 15 and 30 min. Based on these studies, 20:80 PLA/ENR blend (the strongest increase of G’ during compression molding) and 40:60 PLA/ENR blend (the lowest increase of G’ during compression molding) were modified by POSS additives.

**Figure 3:**
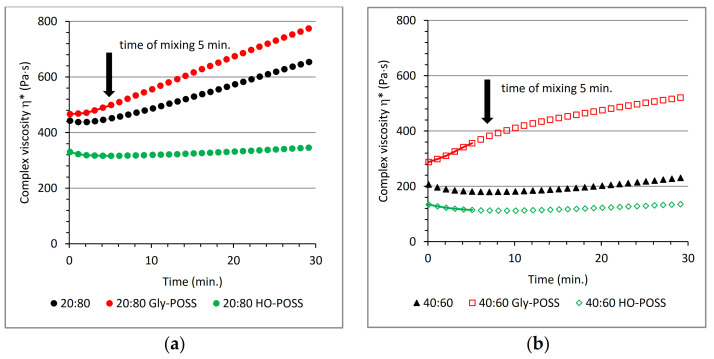
Complex viscosity η* as a function of time at processing temperature 160 °C at constant strain and angular frequency, equivalent of 1 s^−1^ oscillation strain rate for POSS modified 20:80 PLA/ENR (**a**) and 40:60 PLA/ENR blend (**b**).

The influence of POSS, the third component of PLA/ENR blend, on the viscoelastic properties as a function of time at processing temperature 160 °C was analyzed (Figure 3).

The opposite effect of both, HO-POSS and Gly-POSS, on the complex viscosity η* and viscoelastic properties was observed (Figure 3). The incorporation of HO-POSS reduced the complex viscosity of both 20:80 and 40:60 PLA/ENR blends. HO-POSS acted slightly as a plasticizing additive. The presence of isobutyl groups in HO-POSS structure was probably responsible for the lower values of melt viscosity as compared with neat PLA/ENR blends. Other authors [40,45], also observed the plasticizing effect of some POSS structures containing alkyl groups leading to decrease of the melt viscosity of PLA. Moreover, the slightly increase of the η* after 30 min was observed for HO-POSS modified PLA/ENR blends in comparison with not modified PLA/ENR blend. The increases of the η* after 30 min were respectively: ∆η* = 15.5 Pa⋅s for 20:80 PLA/ENR/HO-POSS; ∆η* = 23.4 Pa⋅s for 40:60 PLA/ENR/HO-POSS; ∆η* = 216.4 Pa⋅s for 20:80 PLA/ENR; ∆η* = 50.8 Pa⋅s for 40:60 PLA/ENR. The addition of HO-POSS only slightly influenced on the values of storage shear modulus G’ during 30 min. It suggests that the isobutyl groups linked to the same silicon atom were able to cover the hydroxyl groups reducing its ability to form interactions with other functional groups.

The formation of covalent bonds or hydrogen bonds usually leads to increase of dynamic mechanical properties as it took place in case of Gly-POSS modified PLA/ENR blends. After the incorporation of Gly-POSS into 40:60 PLA/ENR blend the stronger increase of G’ was observed due to self-curing effect. The ∆G’ values after 30 min were respectively: ∆G’= 4 kPa for neat PLA/ENR; ∆G’= 20 kPa for 40:60 PLA/ENR/Gly-POSS. Other authors [43] also reported that epoxy functional groups of POSS particles were able to react with the functional groups present at the end of polyester chains. For PLA/ENR/Gly-POSS blends crosslinking occurred together with the degradation of PLA phase. Higher amount of PLA in 40:60 PLA/ENR blend after the degradation generated higher concentration of the hydroxyl groups. The reaction with Gly-POSS was facilitated as compared with the 20:80 PLA/ENR blend of lower PLA amount in similar volume of the blend.

**Figure 4:**
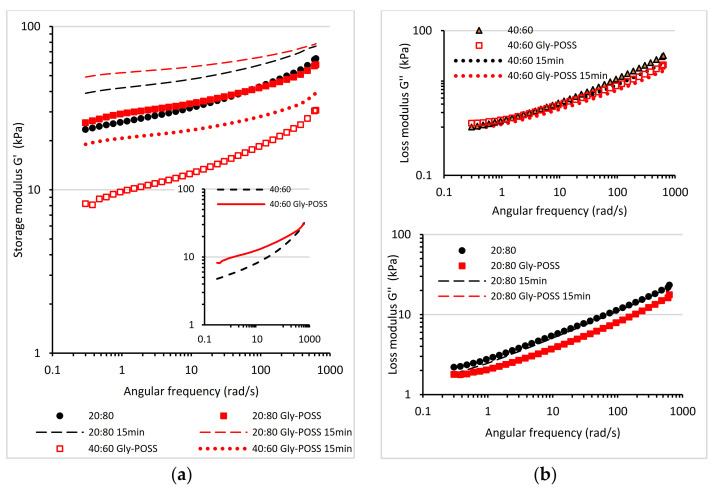
Influence of compression molding during 15 min at 160 °C on the viscoelastic properties of molten PLA/ENR/Gly-POSS blends. Storage shear G’ modulus (**a**); loss shear G’’ modulus (**b**).

**Figure 5:**
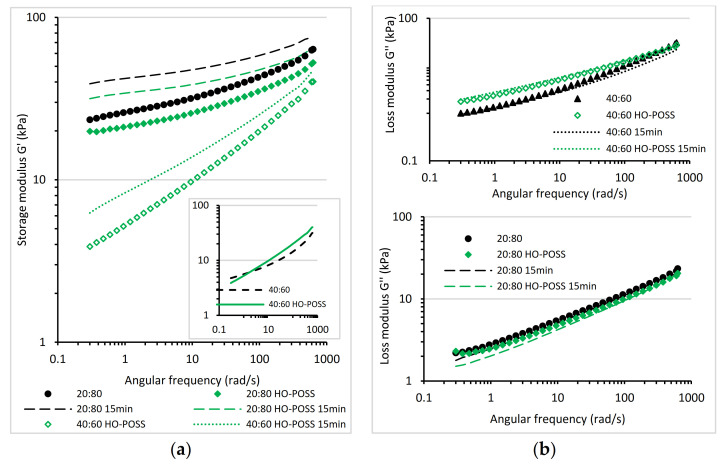
Influence of compression molding during 15 min at 160 °C on the viscoelastic properties of molten PLA/ENR/HO-POSS formulations. Storage shear G’ modulus (**a**); loss shear G’’ modulus (**b**).

To estimate the influence of Gly-POSS and HO-POSS on the viscoelastic behavior of the PLA/ENR blend the frequency sweep tests at 160 °C for freshly prepared blend and for the material compressed molded for 15 min were measured (Figure 4 and Figure 5).

For the freshly mixed 20:80 PLA/ENR blend, the incorporation of Gly-POSS had no significant influence on the values of the storage shear modulus G’ registered as a function of angular frequency (Figure 4). Compression molding of 20:80 PLA/ENR/Gly-POSS for 15 min at 160 °C led to the formation of chemical bonds between Gly-POSS functionalities and PLA and ENR rubber resulting in higher values of the G’ (red dashed line) in comparison with 20:80 PLA/ENR (black dashed line). The incorporation of Gly-POSS into 40:60 PLA/ENR resulted in enhancement of the storage shear modulus G’ of molten blend even before the compression molding. Furthermore, the thermal treatment in press during 15 min caused additional increase of G’ values for 40:60 PLA/ENR/Gly-POSS. The significant enhancement of the value G’ observed after 15 min of compression molding for Gly-POSS modified PLA/ENR blends indicated that Gly-POSS particles can be the functional additive promoting self-curing of PLA/ENR blends. Slightly lower values of loss shear modulus G’’ were observed after incorporation of Gly-POSS into PLA/ENR blends.

Opposite effect was caused by HO-POSS addition into 20:80 PLA/ENR blend, the values of the storage shear modulus G’ were lower for whole studied angular frequency range (Figure 5). The decrease of G’ modulus after incorporation of HO-POSS to 40:60 PLA/ENR was observed only at the low values of angular frequency, less than 1 rad s^−1^. At higher frequency, the presence of HO-POSS led to slight increase of storage shear modulus G’ as compared with neat 40:60 PLA/ENR. The compression molding for 15 min at 160 °C caused the increase of storage shear modulus G’ of both 20:80 and 40:60 PLA/ENR/HO-POSS blends but the G’ values were much lower than that observed for neat PLA/ENR blends. HO-POSS did not participated in curing reactions between PLA and ENR phase. Moreover, the presence of HO-POSS slowed the curing reactions during compression molding of the PLA/ENR blend, indicating that HO-POSS prevented the degradation of PLA.

### 3.2. Morphology of PLA/ENR Blends and Their Dynamic Mechanical Properties at Ambient Temperature

The mechanical properties of the dynamic vulcanized thermoplastic/elastomer blends at ambient temperature usually depends on the formulation of the blend and ratio of both components as well as the morphology of the blend.

**Figure 6:**
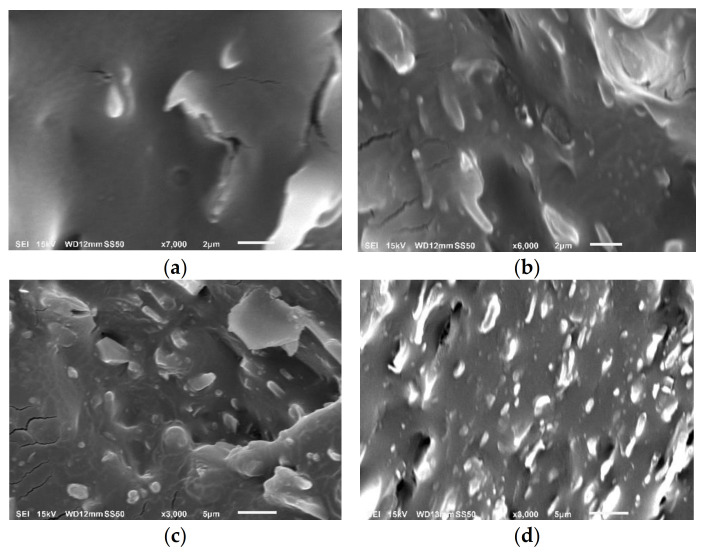
SEM images of 20:80 PLA/ENR (**a**); 30:70 PLA/ENR (**b**,**c**); 40:60 PLA/ENR (**d**).

The SEM analysis (Figure 6) showed phase separation and immiscibility of both phases PLA and ENR in the blend. For both, the 20:80 and 30:70 PLA/ENR blends, the domain-like morphology was formed. The PLA droplets dispersed throughout the elastic ENR matrix were observed. Furthermore, the increase of incorporated PLA amount resulted in changes of the size of the PLA droplets. The diameter of PLA domains in 20:80 PLA/ENR blend was in the range of 1.5–5 µm. Much larger PLA domains (2–6 µm) were observed in case of 30:70 PLA/ENR blend leading to the reduction of the interphase contact between both components of the blend. The highest amount of PLA introduced into blend led to the formation of more co-continuous morphology. The elongated and interconnected shape of PLA dispersed phase was observed. Additionally, the size of these elongated PLA domains was in the range of 1–5 µm. The domains with size larger than 5 µm were observed occasionally. The changes in the morphology of the PLA/ENR blend influenced on the viscoelastic behavior of the material at ambient temperature.

**Figure 7:**
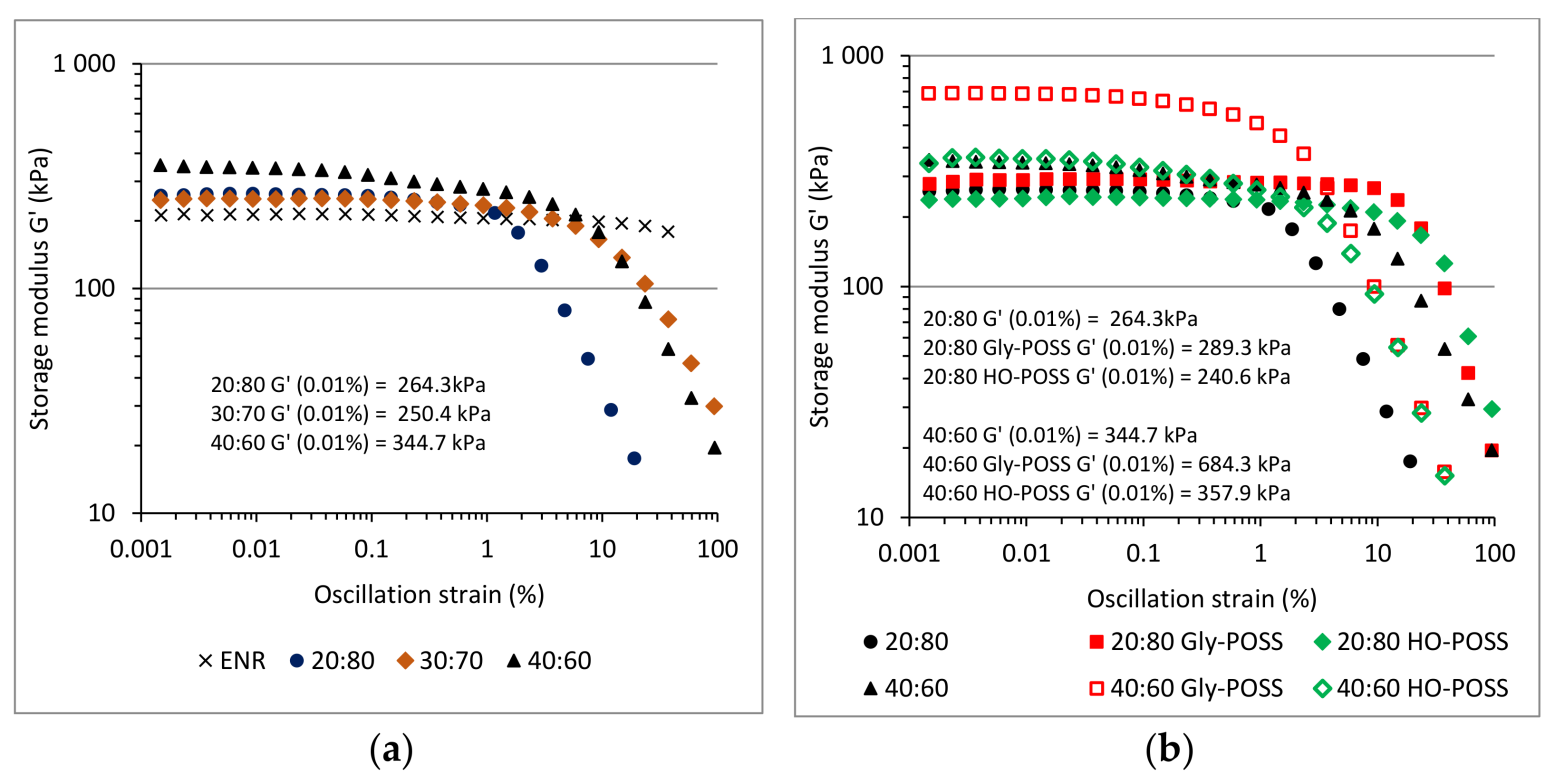
Storage shear modulus G’ as a function of oscillation strain at angular frequency of 10 rad s^−1^ (1.59 Hz) at 30 °C for ENR/PLA (**a**) and POSS modified blends (**b**).

The incorporation of PLA provided significant increase of the storage modulus G’ as compared with neat ENR rubber (Figure 7). The reinforcing effect resulted from the presence of more rigid, thermoplastic phase and it was observed for all blends formulations. The higher ratio of PLA in PLA/ENR blend slightly reduced the values of storage shear modulus G’ due to different morphology of the 30:70 PLA/ENR blend. The less homogenous location and larger size of PLA domains dispersed throughout the elastic ENR matrix led to weaker interfacial adhesion between two components of the blend resulting in the slightly lower values of the storage modulus G’ as compared with 20:80 PLA/ENR blend. The strongest increase of the storage shear modulus G’ was observed for the blend with the highest PLA loading. Moreover, the higher amount of PLA influenced on the range of linear viscoelastic region, in which the values of G’ modulus were strain independent. The incorporated PLA increased strain stability of the blend. For the 30:70 and 40:60 PLA/ENR blends, the storage modulus G’ decreased drastically at the higher range of oscillation strain, more than 10%.

The incorporation of trisilanolisobutyl-POSS (HO-POSS) and glycidyl-POSS (Gly-POSS) nanoparticles, as a third component of PLA/ENR blends, additionally influenced on viscoelastic behavior at ambient temperature (Figure 7). Different effects were observed due to various functional groups of incorporated POSS particles. The glycidyl Gly-POSS enhanced the dynamic mechanical response of PLA/ENR blends leading to the increase of the storage shear modulus G’, especially in case of the blend with higher 40:60 PLA/ENR ratio. As it was pointed by discussion in previous section the grafting of Gly-POSS particles to the PLA polymeric chains and further the reaction of this hybrid grafted structures with functional epoxy groups of ENR rubber led to the formation of crosslinks between two phases. The enhanced interphase interactions increased stiffness of the material after cooling at ambient temperature. Effect was much stronger in case of 40:60 PLA/ENR due to higher amount of PLA in the same volume of the blend. Opposite, the HO-POSS decreased the values of the storage shear modulus G’ of 20:80 PLA/ENR/HO-POSS blend and only slightly increased the G’ modulus at higher PLA concentration (40:60 PLA/ENR/HO-POSS). The presence of isobutyl groups in the structure of HO-POSS attached to the same Si atom restricted accessibility to hydroxyl HO- group reducing possibility of reaction with functional groups of PLA and ENR leading to less cured PLA/ENR blend. Moreover, the higher affinity of isobutyl groups to ENR rubber phase caused a slightly plasticizing effect, reducing the values of storage modulus, especially at low concentration of PLA in the PLA/ENR blend.

**Figure 8:**
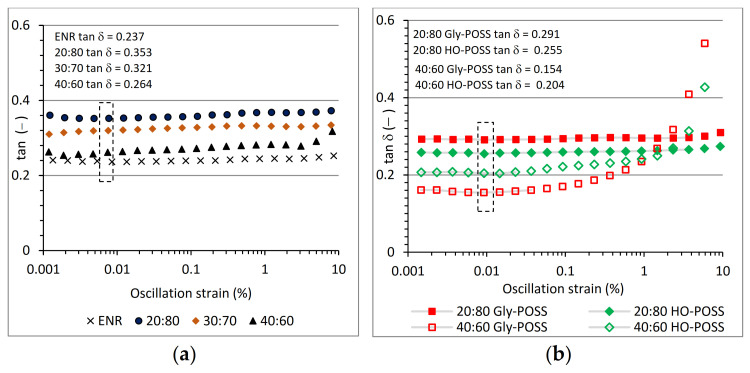
Damping properties, the loss tan δ as a function of oscillation strain at 30 °C and angular frequency of 20 Hz for various PLA/ENR (**a**) and PLA/ENR/POSS formulations (**b**).

The influence of incorporated PLA on the damping properties of PLA/ENR blends were measured as a function of oscillation strain at 20 Hz (Figure 8).

The presence of more rigid PLA caused stronger energy dissipation leading to higher values of loss tan δ for PLA/ENR blends in comparison with neat ENR (Figure 8). As the amount of PLA in ENR/PLA increased the values of loss tan δ decreased. This effect is probably caused by the changes in blend morphology and improvement of interfacial bounding via formation of chemical bonds between functional groups of ENR rubber and end chain groups formed by the chain scission of PLA during processing. At low PLA concentration the dispersed PLA formed separated droplets immersed in elastic ENR matrix. The morphology refrainment to more co-continuous at higher concentration of PLA and improvement of interfacial bounding led to changes in damping properties of the material. Reduction of the loss tan δ was also observed for the 40:60 PLA/ENR and 20:80 PLA/ENR blends modified with both POSS as compared with neat PLA/ENR blend. Although, it should be marked that for the blend with higher amount of incorporated PLA the application of POSS reduced the range of oscillation deformation in which the values of the loss tan δ were linear.

**Figure 9:**
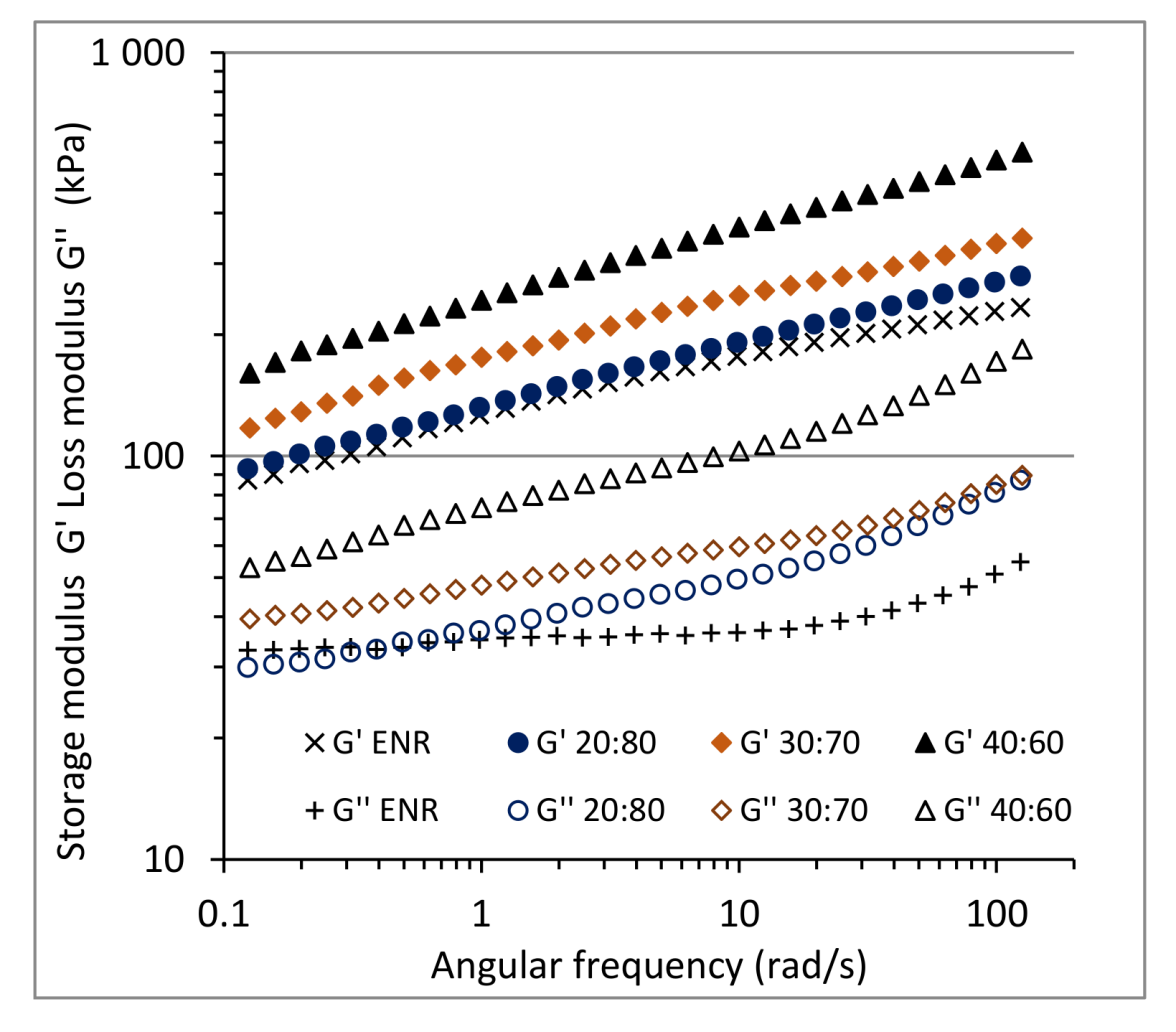
Storage shear modulus G’ and loss shear modulus G’’ as a function of angular frequency at 30 °C for PLA/ENR blends.

Figure 9 displays the evolution of the storage shear modulus G’ and loss shear modulus G’’ as a function of angular frequency for PLA/ENR blends. 

As we expected the incorporation of PLA generated strong reinforcing effect leading to significant increase of the storage shear modulus G as compared with neat ENR rubber. For 20:80 PLA/ENR blend at the short relaxation time (high values of angular frequency ω) the second PLA phase acted as a reinforcing additive, but effect was not as significant as observed for the higher amount of PLA incorporated into blend. At low frequency, the viscoelastic behavior and the values of storage and loss shear modulus of 20:80 PLA/ENR blend were like these observed for neat ENR. In case of 20:80 PLA/ENR blend the presence of PLA did not restricted the relaxation of the material at long time (low values of angular frequency ω). Especially at high frequency, the addition of PLA caused stronger dissipation of energy leading to increase of the values of loss shear modulus G’’ in comparison with neat ENR.

**Figure 10:**
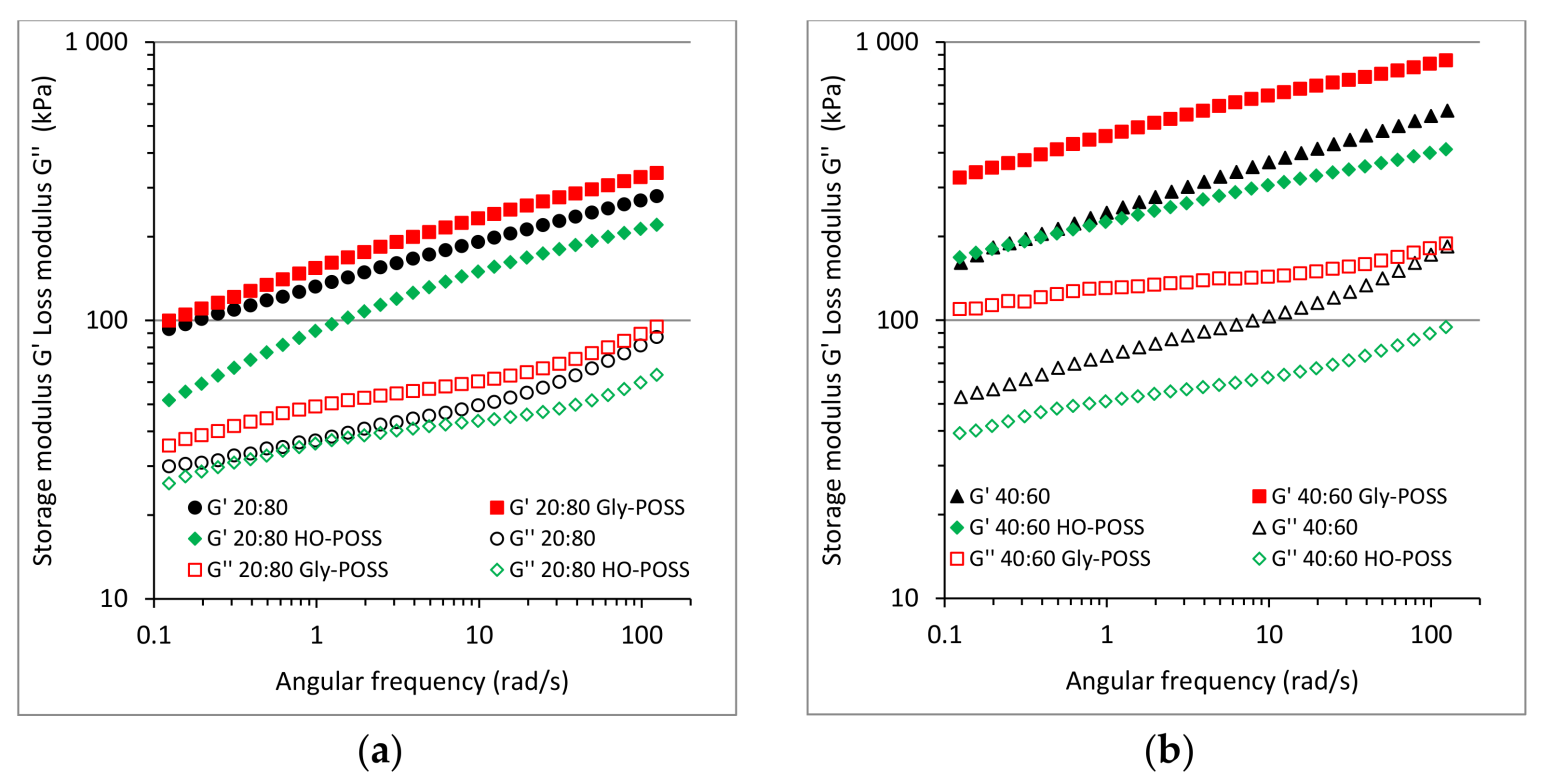
Influence of POSS on the storage shear modulus G’ and loss shear modulus G’’ as a function of angular frequency at 30 °C for 20:80 PLA/ENR (**a**) and 40:60 PLA/ENR (**b**) blend.

The incorporation of both POSS changed the viscoelastic behavior of PLA/ENR blends as showed at Figure 10.

Two factors should be taken under consideration when analyzing the influence of both silsesquioxanes. First, the addition of POSS can change the interfacial tension between both components influencing on the melt blending process during preparation of the blend. Second the presence of active functional groups in POSS moieties allows to form chemical bonds between both components or inside both phases during compression molding. Some authors [40,44,45] reported that various POSS can act as a plasticizing additive for PLA. Consequently, we also reported similar influence and plasticizing effect of HO-POSS for the blend with dominated rubber phase. The incorporation of HO-POSS decreased the values of the storage shear modulus G^’^ of PLA/ENR blends. Conversely, the Gly-POSS significantly enhanced the values of the storage shear modulus G^’^. As we previously described during preparation of the blend in presence of Gly-POSS the chemical bonds were formed leading to higher stiffness of the blend. Therefore, the storage shear modulus G^’^ increased.

### 3.3. Influence of POSS Modification on the Glass Transition Temperature and Thermal Resistance of the Material

**Figure 11:**
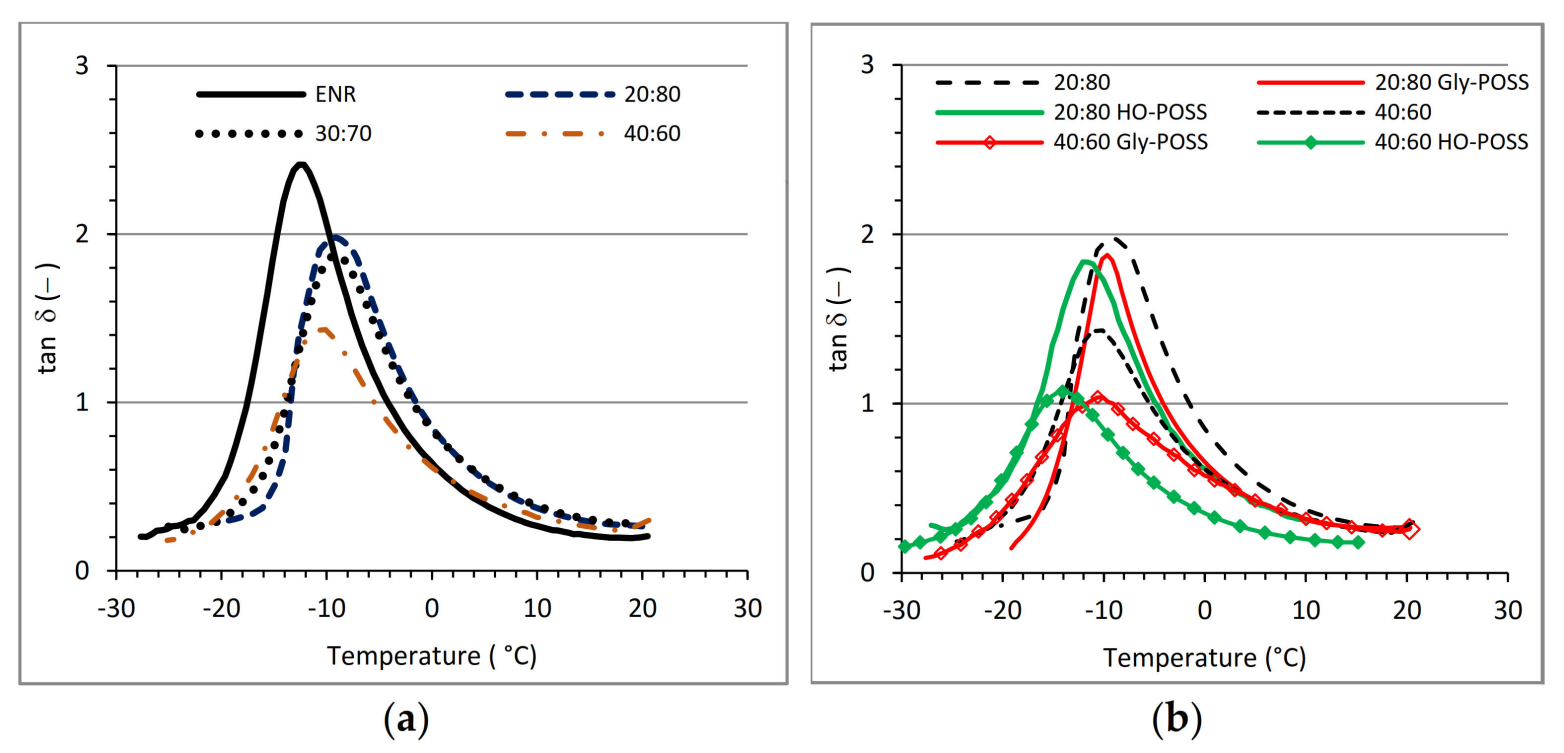
Loss tan δ (−) as a function of temperature (°C) for PLA/ENR (**a**) and POSS modified blends (**b**).

The values of the glass transition temperatures T_g_ of the different blend formulations are shown in Table 1 and Figure 11. The incorporation of second PLA phase slightly shifted the glass transition temperature T_g_ of ENR phase towards higher values of temperature. Moreover, the values of loss tan δ decreased in presence of PLA phase. Further the application of POSS particles as a third component of the blends influenced on the behavior of blend in glass transition region. The lower values of glass transition temperatures T_g_ for PLA/ENR/HO-POSS blends could be additional evidence for the plasticizing influence of that POSS on ENR phase due to presence of isobutyl groups and liquid-like structure. This effect was not observed for Gly-POSS modified blends, the glass transition T_g_ were almost similar to these observed for not modified blends with similar ratio of PLA:ENR. The incorporation of POSS decreased the values of loss tan δ confirming the presence of interphase interactions between components of the blend.

Glass transition temperatures T_g_ of PLA phase in blends were indicated using DSC method (Table 1). The changes in blend formulation, higher ENR to PLA ratio (20:80 PLA/ENR) shifted T_g_ of PLA phase towards lower values of temperature. As the amount of PLA in blend increased the T_g_ of PLA phase became similar to T_g_ of neat PLA. No significant effect of POSS on T_g_ of PLA was observed. It should be mentioned that the DSC curves of PLA/ENR blend showed no cold crystallinity and melting peaks crystalline phase of PLA and the degree of crystallinity for neat PLA calculated based on the enthalpy of cold crystallization and melting enthalpy was relatively low: χ_C_ = 1.13%.

**Figure 12:**
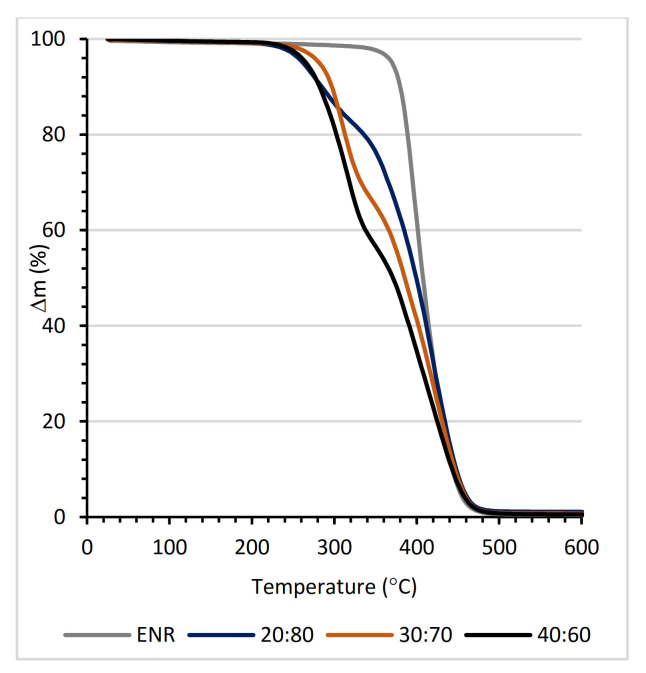
TGA curves of ENR and PLA/ENR blends.

It can be seen from TGA analysis (Figure 12, Table 2) that neat ENR rubber showed single degradation peak, at around 418 °C. Similar thermal stability for ENR rubber was reported by other authors [27]. PLA/ENR blends showed two thermal degradation steps, first due to the weight loss of PLA and second to the ENR. Zheng et al. [32] also observed similar behavior for PLA/ENR blends. Moreover, the temperature of maximum weight loss of PLA phase in PLA/ENR blends is lower than for PLA pellets (Table). The higher content of ENR phase in TPVs increased the thermal stability shifting the temperature of 5% weight loss T_5%_. It should be underlined the stabilizing effect of POSS additives on the thermal stability of created TPVs material. Both incorporated POSS particles increased the temperature of 5% weight loss T_5%_ and the temperature of the maximum weight loss T_max_^PLA^ of PLA phase. Other authors also found that POSS particles can enhance the thermal stability of polymers [34,35].

### 3.4. Tensile Properties of the Blends—Changes in Mechanical Strength after Composting in Soil

Table 3 summarizes the results of tensile strength measurements before and after composting of the material during one month in soil with specific characteristics such as pH, humidity, temperature. The reinforcing effect of incorporation of PLA on the PLA/ENR blend was observed. The higher values of tensile strength TS and SE_100_ modulus, were reported (Table 3). The increase of the tensile strength was 450% for 20:80, 728% for 30:70, and 656% for 40:60 PLA/ENR blend, respectively. As the amount of PLA in blend increased the elongation at break E_b_ decreased together with the significant increase of SE_100_. The formation of covalent bonds between PLA and ENR phases reduced the elasticity of the blend, at the same time the strength of the system was enhanced. Strong reduction of the elongation at break E_b_ after the modification of 40:60 PLA/ENR by Gly-POSS was observed together with lower values of tensile strength as compared with blend modified by HO-POSS. This can be the effect of higher crosslink density of the blend. OV-POSS can act as a multifunctional cross-links reducing elasticity of the material. At the same time, additional cross-links caused excessive brittleness of the material and decreased TS values.

Epoxidized natural rubber ENR, even in uncured state is not degradable material in soil. The presence of unsaturated bonds in rubber chains can lead to crosslinking reactions during ageing. That process was observed for neat ENR after one month of disposal of samples in soil. The determined changes in mechanical properties after composting, higher values of SE_100_ modulus and tensile strength together with calculated values of K_c_ indicating the crosslinking reactions of the ENR. The presence of PLA in the blend influenced on the material degradation during the disposal in soil. PLA under this condition can undergo fragmentation and hydrolytic degradation. During disposal in soil the significant diminution in mechanical properties of PLA phase occurred. The higher amount of PLA in the structure of the blend promoted the degradation in soil causing the deterioration of mechanical properties and reduction of K_c_ coefficient values (Table 2). The impact of HO-POSS modification of 40:60 PLA/ENR blend was promising in this case. The clearly lower K_C_ parameter for the HO-POSS blend indicated more intense degradation processes during composting in the soil.

## 4. Conclusions

The incorporation of thermoplastic poly(lactic acid) PLA into epoxidized natural rubber ENR matrix offers the possibility of creating dynamic vulcanized TPVs pro-ecological blends obtained from renewable resources materials. The analysis of the viscoelastic properties of various PLA/ENR formulations at a processing temperature of 160 °C showed that the higher content of PLA in PLA/ENR reduced the complex viscosity η* of the melt system. Furthermore, by the addition of POSS particles it was possible to influence on the viscosity of the melt PLA/ENR. Trisilanolisooctyl-POSS (HO-POSS) reduced complex viscosity η* due to the presence of the seven isooctyl groups and their plasticizing effect. Opposite, the eight glycidyl functional groups of a glycidyl-POSS (Gly-POSS) were able to form interactions with PLA chains leading to higher viscosity of the melt blend.

After of about 5 min of the thermo-oxidative ageing at 160 °C, continuous increase of the complex viscosity and storage shear modulus G’ and the decrease of loss shear modulus were observed for PLA/ENR blends indicating changes in the structure of the material due to self-curing. The end chain groups of PLA, such as carboxyl and hydroxyl groups (-COOH, -OH), that were formed after PLA hydrolysis and chain scission of PLA reacted with functional, oxirane rings of epoxidized natural rubber ENR; in a similar way as it is observed for the curing of the ENR rubber by amino acids or flavonoids containing hydroxyl groups [29]. Moreover, the self-curing effect of PLA/ENR was strengthened in presence of Gly-POSS indicating that opposite to HO-POSS the Gly-POSS particles participated in curing reactions and acted as multifunctional cross-links.

Studies confirmed that it was possible to generate the curing effect of PLA/ENR by typical processing techniques (compression molding) used in rubber industry. This is a great advantage considering the use of this type of material in industrial practice.

The additive to the 40:60 PLA/ENR blend, such as Gly-POSS, led to significant enhance of the storage shear modulus G’ at ambient temperature as measured both in function of oscillation strain and angular frequency. Furthermore, the glass transition temperature T_g_ of ENR phase for Gly-POSS modified PLA/ENR blends were shifted to higher temperature as compared with neat ENR rubber. It is additional confirmation that Gly-POSS particles participated in curing of the PLA/ENR blend, and acted as multifunctional cross-links restricting the mobility of polymeric chains. Both incorporated POSS influenced on the damping properties of PLA/ENR blends at 30 °C leading to the decrease of loss factor tan δ measured as a function of oscillation strain.

The presence of solid PLA phase in the blend enhanced the static mechanical properties of the material, the tensile strength increased in comparison to virgin ENR rubber. At the same time, the PLA/ENR material retained its elasticity and acceptable values of elongation at break E_b_. The obtained values of the tensile strength TS and elongation at break E_b_ for 40:60 PLA/ENR/Gly-POSS blend indicated higher stiffness of the material as compared with an unmodified blend.

The PLA phase influenced on the degradation of the material during composting leading to the significant diminution in mechanical properties of the material. The composting coefficient K_C_ was significant lower for HO-POSS modified 40:60 PLA/ENR blend confirming that the presence of HO-POSS promoted the degradation of the material in soil. The PLA/ENR POSS modified blends described in this paper, especially 40:60 PLA/ENR/HO-POSS blend due to its enhanced static mechanical properties together with higher affinity to compostability, are suitable for many application. In particular, PLA/ENR TVP blends can be used in areas where the final product requires a higher content of bio-renewable components.

## Figures and Tables

**Table 1:** Glass transition temperature T_g_ of ENR and PLA phase for PLA/ENR blends.

	T_g_^ENR^, (°C)	Tan δ at T_g_^ENR^, (-)	T_g_^PLA^, (°C) (*)
ENR or PLA	−12.64	2.41	55.21
20:80	−9.13	1.98	51.2
20:80 Gly-POSS	−9.64	1.88	52.32
20:80 HO-POSS	−12.06	1.84	51.23
30:70	−9.62	1.88	52.91
40:60	−10.11	1.43	54.48
40:60 Gly-POSS	−10.08	1.04	55.39
40:60 HO-POSS	−14.10	1.07	54.79

**Table 2:** Temperature of 5% weight loss T_5%,_ temperature of maximum weight loss of PLA phase T_max_^PLA^, temperature of maximum weight loss of ENR phase T_max_^ENR^.

	T_5%_, (°C)	T_max_^PLA^, (°C)	T_max_^ENR^, (°C)
PLA pellets	328	350	-
ENR	367	-	418
20:80	259	287	438
20:80 Gly-POSS	280	307	435
20:80 HO-POSS	292	339	439
30:70	277	324	443
40:60	265	330	443
40:60 Gly-POSS	292	340	448
40:60 HO-POSS	286	337	439

**Table 3:** Tensile mechanical properties of PLA/ENR blends before and after composting and the value of composting coefficient K_C._

	SE_100_, (MPa)	TS, (MPa)	TS^*^, (MPa)	E_b_, (%)	E_b_ *, (%)	K_C_, (−)
ENR	0.26 ± 0.01	0.50 ± 0.04	0.68 ± 0.05	399 ± 31	369 ± 73	1.26 ± 0.05
20:80	0.95 ± 0.19	2.25 ± 0.22	2.24 ± 0.32	418 ± 87	352 ± 91	0.84 ± 0.04
20:80 Gly-POSS	0.75 ± 0.13	2.23 ± 0.73	1.73 ± 0.41	360 ± 23	357 ± 31	0.77 ± 0.03
20:80 HO-POSS	1.18 ± 0.20	2.49 ± 0.35	2.05 ± 0.39	247 ± 21	241 ± 42	0.80 ± 0.02
30:70	1.73 ± 0.24	3.64 ± 0.32	3.34 ± 0.22	337 ± 53	281 ± 48	0.76 ± 0.03
40:60	2.42 ± 0.26	3.28 ± 0.16	3.00 ± 0.27	179 ± 30	133 ± 28	0.68 ± 0.02
40:60 Gly-POSS	-	3.24 ± 0.42	2.33 ± 0.31	48 ± 13	38 ± 24	0.56 ± 0.03
40:60 HO-POSS	5.46 ± 0.38	5.12 ± 0.47	3.86 ± 0.39	119 ± 15	48 ± 17	0.30 ± 0.02

* values for the samples measured after one month of composting

## Data Availability

Data is contained within the article.

## Supplementary Materials

The following are available online at https://www.mdpi.com/article/10.3390/ma14102686/s1, Figure S1: Influence of thermal treatment during 15 and 30 min of compression molding at 160 °C on the viscoelastic properties of neat ENR rubber, the storage shear modulus G’; Figure S2: Influence of thermal treatment during 15 and 30 min of compression molding at 160 °C on the viscoelastic properties of neat ENR rubber, the loss shear modulus G.
