# Supplementary material for: Viscoelastic Properties of Epoxidized Natural Rubber/Poly(lactic acid) PLA/ENR Blends Containing Glycidyl-POSS and Trisilanolisooctyl-POSS as Functional Additives
Source: Materials (Basel). 2021 May 20;14(10):2686. doi: 10.3390/ma14102686 (PMC8160644; doi:10.3390/ma14102686)
Supplement: Supplementary file 1 [file materials-14-02686-s001.zip › materials-1201630-supplementary.pdf]

Viscoelastic properties of epoxidized natural rubber/ poly(lactic acid) PLA/ENR blends containing glycidyl-POSS and trisilanolisooctyl-POSS as functional additives.

Magdalena Lipińska[1,*], Klaudia Toczek[2] and Magdalena Stefaniak[3]

[1)] Lodz University of Technology, Institute of Polymer and Dye Technology; magdalena.lipinska@p.lodz.pl
[2)] Lodz University of Technology, Institute of Polymer and Dye Technology; klaudia.toczek@dokt.p.lodz.pl
[3] Lodz University of Technology, Institute of Polymer and Dye Technology; magdalenastefaniak95@gmail.com
* Correspondence: magdalena.lipinska@p.lodz.pl ;

Supporting Information:

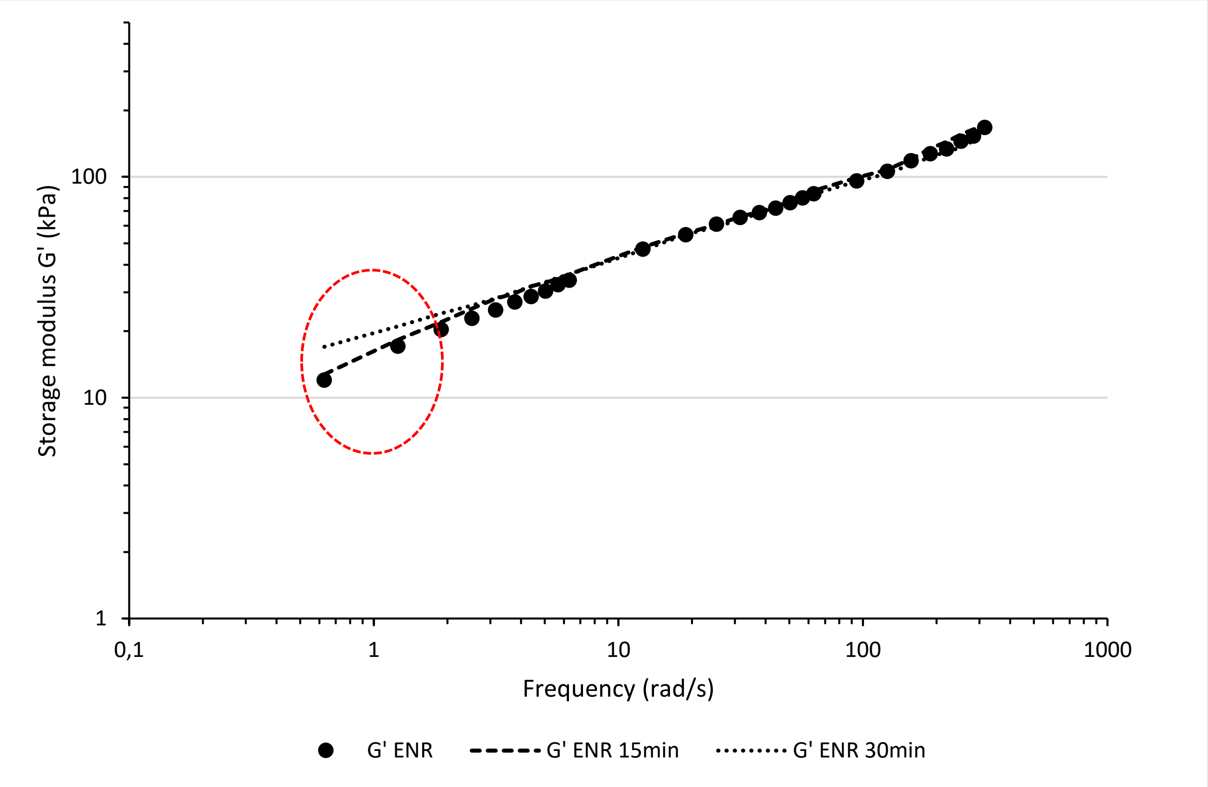

Figure S1: Influence of thermal treatment during 15 and 30 min. of compression molding at 160°C on the viscoelastic properties of neat ENR rubber, the storage shear modulus G’

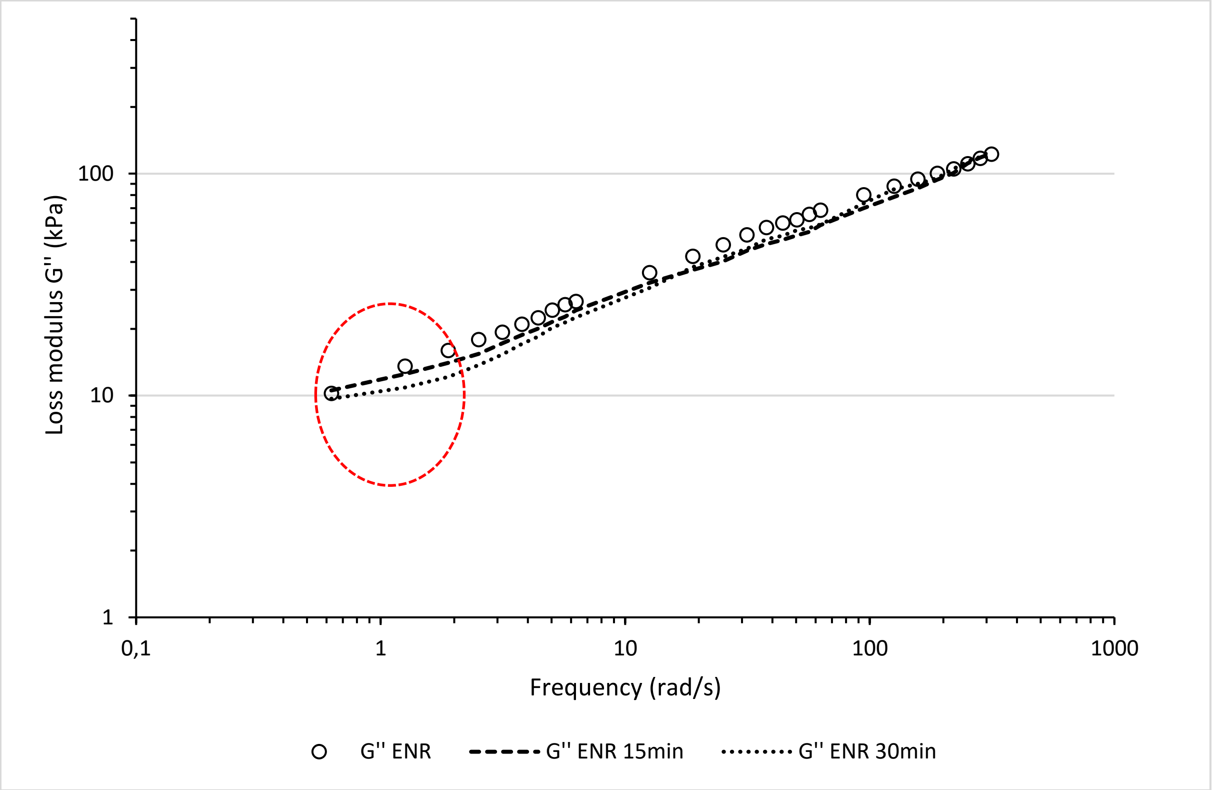

Figure S2: Influence of thermal treatment during 15 and 30 min. of compression molding at 160°C on the viscoelastic properties of neat ENR rubber, the loss shear modulus G''

No significant changes in viscoelastic behavior and the values of storage shear modulus G' of neat ENR rubber due to the thermal treatment during 15 min. and 30 min. were observed for the higher frequency. The slightly higher values of the storage shear modulus G' were observed for the sample thermally treated in hydraulic press during 30 min. at low values of frequency, lower than 1 rad·s^{-1}. Similarly, no significant effect of thermal treatment in hydraulic press was observed in case of loss shear modulus G'' for the higher frequency. At lower frequencies (less than 1 rad·s^{-1} slightly lower values of G'' were indicated.
